# Understanding the impacts of health information systems on patient flow management: A systematic review across several decades of research

**DOI:** 10.1371/journal.pone.0274493

**Published:** 2022-09-12

**Authors:** Quy Nguyen, Michael Wybrow, Frada Burstein, David Taylor, Joanne Enticott

**Affiliations:** 1 Department of Human-Centred Computing, Faculty of Information Technology, Monash University, Melbourne, Australia; 2 Office of Research and Ethics, Eastern Health, Melbourne, Australia; 3 Monash Centre for Health Research and Implementation, Monash University, Melbourne, Australia; University of Hong Kong, HONG KONG

## Abstract

**Background:**

Patient flow describes the progression of patients along a pathway of care such as the journey from hospital inpatient admission to discharge. Poor patient flow has detrimental effects on health outcomes, patient satisfaction and hospital revenue. There has been an increasing adoption of health information systems (HISs) in various healthcare settings to address patient flow issues, yet there remains limited evidence of their overall impacts.

**Objective:**

To systematically review evidence on the impacts of HISs on patient flow management including what HISs have been used, their application scope, features, and what aspects of patient flow are affected by the HIS adoption.

**Methods:**

A systematic search for English-language, peer-review literature indexed in MEDLINE and EMBASE, CINAHL, INSPEC, and ACM Digital Library from the earliest date available to February 2022 was conducted. Two authors independently scanned the search results for eligible publications, and reporting followed the PRISMA guidelines. Eligibility criteria included studies that reported impacts of HIS on patient flow outcomes. Information on the study design, type of HIS, key features and impacts was extracted and analysed using an analytical framework which was based on domain-expert opinions and literature review.

**Results:**

Overall, 5996 titles were identified, with 44 eligible studies, across 17 types of HIS. 22 studies (50%) focused on patient flow in the department level such as emergency department while 18 studies (41%) focused on hospital-wide level and four studies (9%) investigated network-wide HIS. Process outcomes with time-related measures such as ‘length of stay’ and ‘waiting time’ were investigated in most of the studies. In addition, HISs were found to address flow problems by identifying blockages, streamlining care processes and improving care coordination.

**Conclusion:**

HIS affected various aspects of patient flow at different levels of care; however, how and why they delivered the impacts require further research.

## 1. Introduction

Patient flow refers to the progressive movement of patients through different units or departments of the care setting. The aim of patient flow management is to provide safe and efficient patient care while assuring the best use of resources [1]. Hospitals around the world have undertaken several efforts and strategies to tackle patient flow problems and to provide high-quality care at the right time and right place. Meanwhile, there is an extensive stream of research reporting methods and interventions addressing patient flow problems. A recent umbrella review [2] found that over 25 different interventions have been used by hospitals around the world to solve the overcrowding issues in the emergency department (ED). However, previous studies focused primarily on interventions for a single, isolated hospital unit or ward with ED being the most frequently mentioned [3, 4]. While many systematic reviews related to patient flow interventions have been done, a summary of these systematic reviews shows that most of these reviews have focused on traditional, non-IT interventions such as triage, streaming, and fast track. Systematic reviews on using health information systems (HISs) to tackle patient flow problems exist; however, they are often limited to a single specific system, such as computer provider order entry (CPOE) system [5]; methods such as computer simulation modelling [6]; or measures such as length of stay (LOS) [7].

HISs have been adopted by health providers to improve patient flow in various healthcare settings. For example, in emergency care, the automatic push notification system was used to address ED congestion, reduce LOS, and decrease patient load by providing updated information and improving patient navigation [8]. Dashboard systems were adopted to coordinate ambulance services and improve access to emergency services across multiple hospitals [9]. HISs provides data about ED visits which were used to create a robust prediction about hospital admissions and increase logistical efficiency [10]. In addition, Blaya et al. [11] investigated the use of HISs in improving access to laboratory results and the quality of care. These are a few examples illustrating the impacts of HISs on patient flow management.

In recent patient flow research, it has been suggested that utilising advanced data analytics techniques for patient flow management can be achieved by adopting HISs. For example, Rutherford et al. [12] claim that data analytics is essential in achieving improvement in systematic-wide flows through its capabilities in matching patient demand and hospital supply. Real-time demand capacity has been successfully implemented in many healthcare organisations to predict and match supply and demand [13]. Similarly, Berg et al. [14] called for a shift in the research paradigm from predicting and controlling to analysing and managing to achieve better flow outcomes. This can be done through the application of information technology in analysing data to proactively manage patient flows. Despite the rich tradition of inquiry in research about the use of HISs in patient flow management, to date, to the best of our knowledge, no systematic review has been conducted to assess the impacts of a broad range of HISs on patient flow management, highlighting an evidence gap in the literature. Therefore, a systematic review of this topic will provide more complete insights as to how HISs have been adopted for and impacted patient flow management practice.

## 2. Objectives

This systematic literature review aimed to examine and summarise information from published studies on the use of HISs in healthcare settings to manage and improve patient flows. We are interested in exploring what information systems have been adopted for managing patient flow and solving flow problems such as blockages, delays, and overcrowding, and their effectiveness. We examined studies that focused on department-level (e.g., ED), hospital-wide, and network-wide interventions. Particularly, our objectives are to provide critical analysis on:

- Study characteristics: Chronological and geographical distribution of the studies, study settings, and research designs.
- Study contents: What types and features of information system have been used for patient flow management, their results and effectiveness on patient flow outcomes.

### 3. Research questions

This review addresses the following research questions:

- What HISs have been used for hospitals’ patient flow management?
- What are the impacts of HISs on patient flow outcomes?
- In what ways, have HISs been used to manage patient flow?

## 4. Method

### 4.1 Search strategy

We searched for peer-reviewed journal articles published in English from MEDLINE and EMBASE via Ovid, CINAHL, INSPEC, and ACM Digital Library from the earliest date available to February 2022. In addition, we examined the reference lists of the search results to retrieve further eligible papers. The search was conducted from June 2020 to July 2020 and then re-run in February 2022 before the data extraction process.

With the assistance of a subject librarian, we developed a systematic search strategy for this review (S1 File). To obtain the most comprehensive search results, we employed medical subject headings (MeSH) keywords when they are available in combination with free text keywords from the PICOS framework. We combined the following terms (Table 1) in our search for relevant studies.

**Table 1:** Search terms.

Keyword	Boolean	Additional Keyword
*information system	AND	Patient flow or Hospital flow
Electronic health record or EHR		Patient throughput
Electronic medical record or EMR		Patient journey
Decision support system or DSS		Overcrowding
Business intelligence system or BI system		Access block
Computerised provider order entry system or CPOE		Waiting time
Electronic bed board systems		Length of stay

First, we used the term “information system” (IS) as a general search term and the asterisk (*) because it could include different types of IS used in hospitals such as hospital IS, health IS, healthcare IS, or departmental ISs such as ED IS, intensive care unit (ICU) IS. Acknowledging the fact that hospitals adopt various types of IS with specific terminologies, we also included specific ISs commonly used by hospitals such as electronic health record (or EHR) or electronic medical record (or EMR), decision support systems (or DSS), business intelligence (BI) system, computerised order entry (CPOE) system and electronic bed board systems. In a similar vein, “patient flow” was used as a main keyword together with other synonyms such as “hospital flow”, “patient throughput”, “patient journey”. Common indicators of patient flow management such as length of stay and waiting time were also included in the search. In addition, we used the bibliography of the selected papers to reach further studies. This technique is known as backward snowballing [15].

### 4.2 Eligibility

Table 2 specifies the inclusion and exclusion criteria used in the title and abstract screening process for this review.

**Table 2:** Inclusion and exclusion criteria applied in the screening process.

	Inclusion	Exclusion
Study setting	• Study settings that include, primary, secondary, tertiary hospitals and clinical centres. • Studies investigate the use of IS for patient flow management in hospital units such as ED or ICU were also included for review	• Studied outside these settings
Type of Interventions	• Computerised information systems used by hospitals or clinic centres	• Other interventions • Single medical tools such as a CT scanner or heart measuring device. • Personal Devices
Type of publication	• Articles published in peer-reviewed journals and conferences • Full-text available • English language	• Other types of publication such as: book chapters, reports, non-scholarly publications, reviews • Full-text is not available • Published in other languages

We included studies that described the impacts of HISs that were actually implemented and adopted for managing patient flow or solving patient flow problems. We excluded papers describing prototype systems, systems that were not implemented in practice or papers without real impacts of HISs on patient flow management such as those just reporting simulated results, simulation tests, or prediction models. Studies that focused on measures such as length of stay (LOS), and waiting time for clinical purposes without any relation to or discussion of patient flow management purposes were also excluded from this review.

#### Type of studies

Apart from excluding simulation studies and review papers, we imposed no restrictions on the study’s design or publication date as long as the studies examined the effects of HIS on patient flow management.

#### Participants

We included studies that were conducted in various healthcare settings including teaching hospitals, specialist hospitals and general hospitals (both public and private) and clinical centres. As long as the studies were conducted in these settings, we imposed no restrictions on the number of departments, units or wards involved. We also selected studies that addressed patient flow management at the network level, i.e., between different hospital sites and hospital centres. Studies investigating interventions in services not directly related to patient flow and patient access (such as financial services or insurance) were excluded from our review.

#### Type of intervention

Health information system is a broad concept and hospitals generally adopt and use several types of information systems to manage their operations. In this review, we selected studies that addressed any type of computerised information systems that have been implemented and had impacts on patient flow outcomes. We also excluded paper-based information management systems, personal digital assistance devices, and medical tools such as surgery robots, CT scanners, heart rate measuring devices.

### 4.3 Study selection

To assist the selection of eligible studies for this review, we used the Preferred Reporting Items for Systematic Reviews and Meta-Analyses (PRISMA) guidelines with four key phases [16].

Initially, the first author searched through the pre-identified online databases by using combinations of the keywords to identify related studies. Duplicates were subsequently removed by using a tool called Covidence [17] and manually double checking by the first author. In the second step, two reviewers scanned the abstract of all studies to remove irrelevant or ineligible studies based on the predefined inclusion and exclusion criteria. The remaining studies went into the third step in which two reviewers assessed the full-text studies and further eliminated irrelevant papers. The final phase involved extracting data from included studies. We endeavoured to look for full-text files of the eligible papers in all resources available including using intra-library service to retrieve as many as possible

### 4.4 Data extraction and quality assessment

Information from the papers was extracted in the final list using an electronic data extraction form. Each study was given a unique identification number to ensure a consistent way of identifying studies between the two reviewers. The following data were extracted: authors, journals where the studies were published, year of publication, hospital’s country, the study settings, study objectives, study design, description of the information systems used, factors affecting the adoption of HISs for patient flow management, the effects of HISs on patient flow outcomes, study results, study limitation and research gaps (S4 File, Example of data extraction form).

The GRADE [18] approach was adopted to assess the overall quality level of the evidence based on their design. GRADE approach provides particular useful guidelines for assessing health technology studies with heterogeneous study designs. Using the guidelines, the quality of evidence would be assessed as follows:

- High quality for randomized trial studies without serious limitations
- Low quality for observational studies
- ‘0’ level of quality for studies where quality is not assessable such as expert opinion and studies without objective evidence.

### 4.5 Analytic frameworks

We adopted literature review and expert opinion to develop frameworks that describe types of HISs, their functional capabilities, and associated benefits (Table 3). We also used the conceptual model of Donabedian [19] as a framework for the analysis on patient flow outcomes. Donabedian’s model categorises care quality into three groups: structure, process, and outcomes.

**Table 3:** Analytic frameworks of health information systems.

*Framework*	*Reference*	*Elements*
*Types of HIS*	Expert opinion	• Patient tracking system • Electronic Health Record (EHR) systems • Electronic Medical Record (EMR) systems • Computerised provider order entry (CPOE) systems • Dashboard systems • Workflow management systems • Clinical document management systems Other
*HIS capability*	Expert opinion	• Patient or event tracking • Document management • Order entry • Patient registration • Bed management • Decision support • Discharge management • Patient flow reporting • Prescription management Other
*Patient flow outcomes*	Donabedian model [19]	• Structure: measures related to healthcare centres’ capability to deliver care such facilities, human resource. • Process: measures related interactions between care providers and patients, and how care providers deliver care. • Outcomes: measures related to the end results of the care processes.

## 5. Results

**Fig 1:**
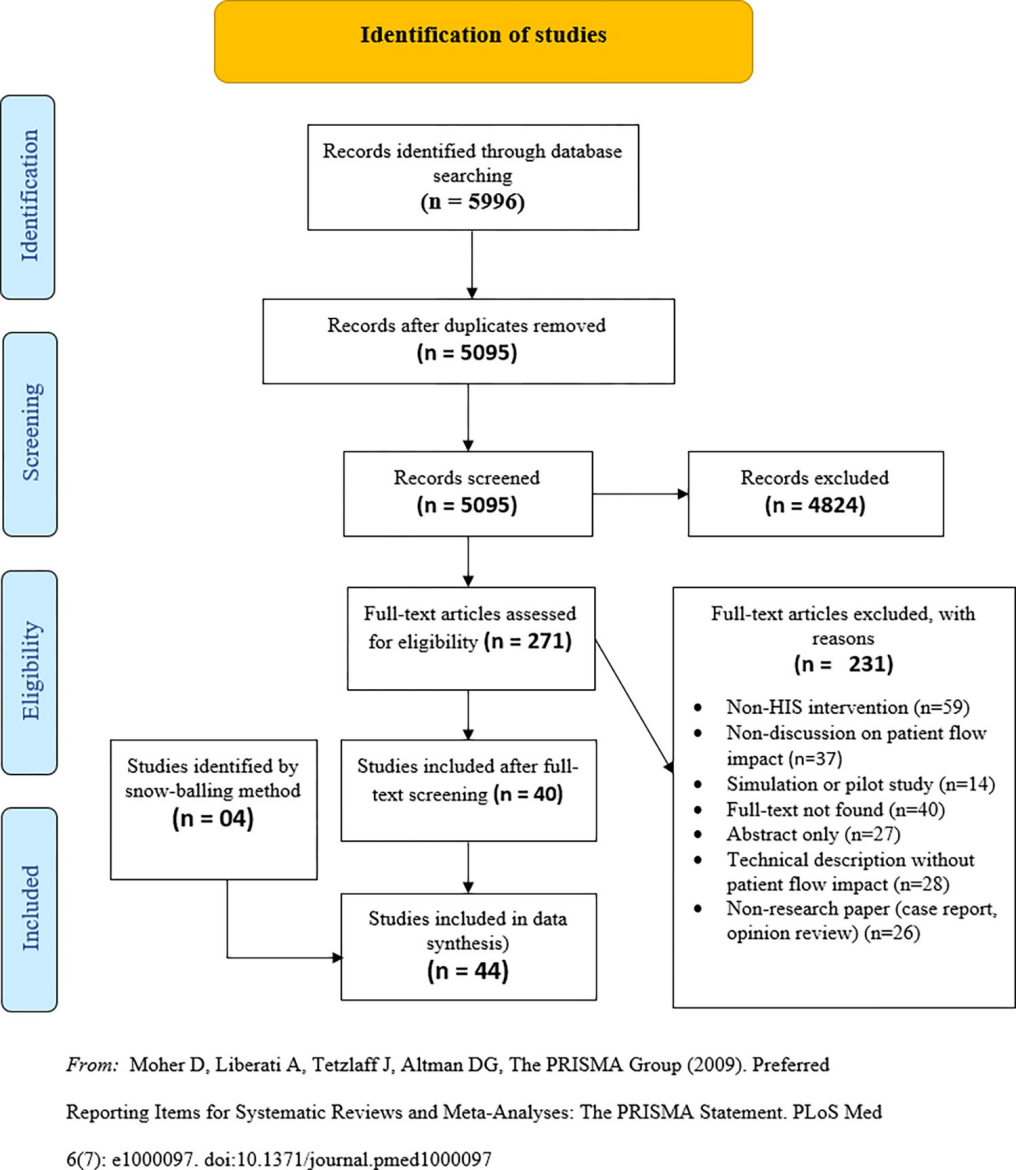
Study screening process. *From*: Moher D, Liberati A, Tetzlaff J, Altman DG, The PRISMA Group (2009). Preferred Reporting Items for Systematic Reviews and Meta-Analyses: The PRISMA Statement. PLoS Med 6(7): e1000097. doi:10.1371/journal.pmed1000097.

The literature search returned 5996 studies and the removal of duplicates reduced the number to 5095. After the first level of screening in which we screened the titles and abstracts and applied the exclusion criteria, 4824 studies were removed. We then proceeded to screen the full-text of 271 studies and 231 of them were excluded. In addition, four studies were added to the final pool through the reverse snowballing technique. Details of the screening process is summarised in Fig 1, following the PRISMA flow diagram [16].

We included 44 studies for our systematic review. The included studies reported mixed impacts of HISs on patient flow management, which can be categorised as follows:

- 33 studies reported positive impacts [9, 20–51]
- 7 studies reported negative impacts [52–58] and 4 studies reported no impacts of ISs [59–62]. However, among the seven studies with negative impacts, two [55, 58] found that the negative effects were temporary and the patient flow measures returned back at pre-implementation baselines.

### 5.1 Types and features of the HIS

The included 44 studies reported the impacts of 17 different types of HIS on patient flow: eight EHR systems, eight EMR systems, seven patient tracking systems, four computerised provider order entry systems (CPOE), three patient flow dashboard systems, three departmental information systems including ED (1) and Radiology (2), and one each for workflow management, admission prediction, documentation management, patient scheduling, medical prescribing, patient discharge management, patient referral management system, bed management, consultation management, clinical information management, and Asthma management. Table 4 summarises details of the study site and publication profile of the included studies (publication year, country and study settings).

**Table 4:** Publication and study site profile of included studies.

*Classification Criteria*	*Variables*	*Frequency (number of studies)*	*%*
*Publication year*	1988–2000	3	6.8%
	2001–2010	13	29.5%
	2011–2020	28	63.6%
*Country*			
	USA	24	54.5%
	Australia	9	20.5%
	Canada	3	6.8%
	South Korea	2	4.5%
	England, Italy, Japan, Portugal, Uganda, Taiwan (one study originated from each)	6	13.6%
*Study setting*			
	Departmental level	22	50%
	*Emergency Department*	*17*	*38%*
	*Radiology Department*	*3*	*7%*
	*Paediatric Department*	*2*	*5%*
	Hospital-wide	18	41%
	Network-wide	4	9%

Research on the application of HISs to patient flow management can be dated back to the 1980s; however, it has gained prominence over the last decade. A majority of included studies were published in the period 2011–2020 (63.6%), compared to 29.5% of the 2001–2010 period and 6.8% of the 1988–2000 period. In addition, most of the studies selected for this review were published in developed countries where their governments have implemented promotional programs to increase the adoption of HISs in the healthcare sector. The number of studies from the USA was the highest with 24 studies, followed by Australia with nine studies. Canada and South Korea contributed three and two studies, respectively. One study was conducted in each of the followings: England, Italy, Japan, Portugal, Uganda, and Taiwan.

**Fig 2:**
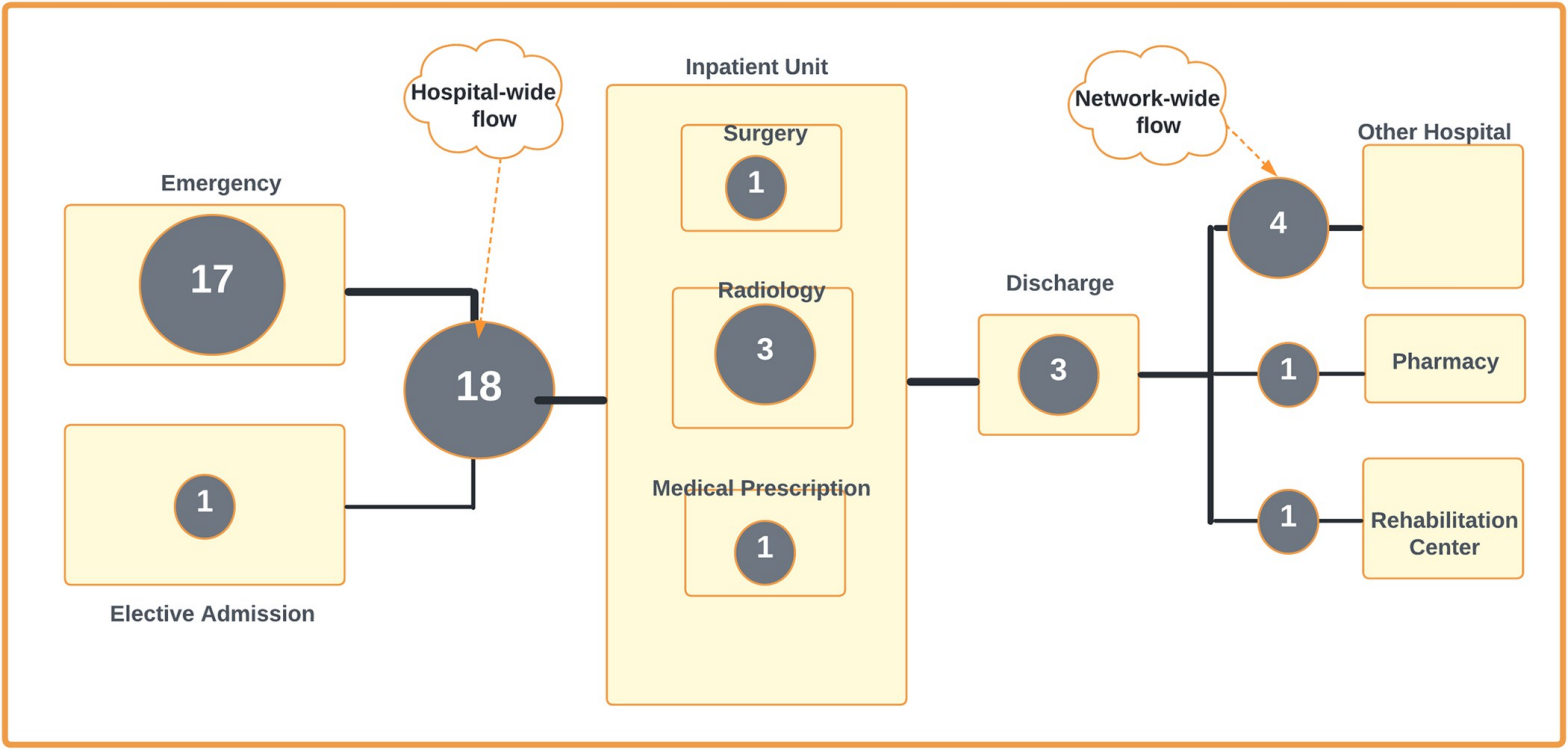
Focused patient flow areas of the reported HISs. The numbers in the circles correspond to the number of relevant studies reviewed.

In terms of settings, 20 of the reviewed studies discussed the impacts of HIS interventions at the department level, while eleven studies addressed hospital-wide level and three studies address network-wide level. Within the department level, 15 studies focused on EDs, three in Radiology and two in Paediatrics. Studies focused on hospital-wide patient flow when they include the coordination between several departments or units. For example, Westbrook et al. [51] discussed the impacts of CPOE on the flow of patients between ED and Pathology departments in Australian hospitals. In addition, we found that four studies described the impacts of HISs on patient flow across hospital networks [9, 31, 39, 42]. Fig 2 depicts where the reported HIS were studied in the care continuum and the number of studies.

Specific functions of the 17 types of HIS that were described in the 44 studies included patient or event tracking (12 studies), clinical documentation management (12 studies), order entry (8 studies), patient registration (3 studies). Bed management, decision support, discharge management, patient flow reporting and prescription management were each included in three studies. Alert, disease detection, picture archiving, staff performance management, referral management, and reminder, were each discussed in one study. Almost all of the included HISs had the capability to integrate data from other systems. Twelve studies did not describe system features. Details of the HIS features and reported benefits are provided in S2 File.

### 5.2 Impacts on patient flow measures

Table 5 provides a summary of how key patient flow measures were grouped into three categories based on Donabedian model [19] and the number of studies that included these measures. Details of the included studies and HISs’ impacts on patient flow measures are provided in S3 File, Characteristics of all included studies and their findings.

**Table 5:** Patient flow measures and the citation number of included studies.

*Type of Measures*	*Detail*	*Citation Number*
***Outcome Measure***		
*Individual outcome*		
	LOS	[9, 22–25, 29, 32, 33, 36, 38, 39, 41, 43, 45, 51–55, 57–62]
	Waiting time	[20–23, 31, 35, 42, 43, 46, 53, 54, 56, 58, 59]
	Treatment time	[22, 23, 44, 58–60]
	TAT	[21, 22, 38, 41, 48, 51]
	LWBS	[33, 54, 56, 61]
	Patient satisfaction	[35, 58, 62]
	Boarding Time	[22, 43, 52]
	Readmission rate	[37]
	Mortality	[32]
*Organisational outcome*		
	Hospital costs	[33, 41, 47, 53]
	Film saving	[41]
	Staff satisfaction	[49]
	Staff stress level	[35]
***Process Outcome***		
	Number of treatment or Medical staff productivity	[31, 41, 55],
	Number of early discharges	[37, 49]
	Guideline Adherence	[60]
	Number of shifts per staff	[56]
	Number of prescriptions	[49]
***Structure outcome***		
	Room utilisation or occupation rate	[27]
	Number of diversions	[33]
	Average number or % of Access block	[27]
	EMS site avoidances	[38]
	ED patients with > = 12 LOS	[33]
	% of AV offload waiting time > 30m	[56]

#### Impacts on outcome measures

Outcomes measures were the most studied measures in the included papers. This is not surprising because health outcomes are the end products of care and the target of health interventions. Studies examined two main types of outcome measures: individual outcomes and organisational outcomes. Almost all studies focused on individual outcome measures in which time-related measures included LOS (25), waiting time (13), treatment time (6), test turnaround time (TAT) (6), and boarding time (2). The effects of HISs on these time related measures were mixed. With regard to LOS, following the use of HISs: 14 studies [22–25, 29, 32, 33, 36, 38, 39, 41, 43, 45, 51] reported a decrease in LOS, 7 studies [9, 52–55, 57, 62] reported an increased LOS and 4 studies [58–61] found no difference. While most of these studies measured LOS in the ED, five studies [25, 39, 41, 43, 45] measured inpatient LOS and two studies [23, 59] reported changes in patient LOS at paediatrics centres. The ED LOS was not consistently defined. ED LOS was defined as the difference between ED exit time and the recorded arrival time [52]. Whereas some studies [22, 59] calculated detailed components which constitute the total LOS including time from arrival to triage, arrival to doctor, doctor to disposition, most other studies just reported the mean LOS.

Similarly, 14 studies reported impacts of HISs on waiting time. The results were mixed with 10 positive changes (reduction in waiting time), 2 negative and one with no statistically significant difference. Waiting time measures included waiting for the doctor, waiting for medical treatment, waiting for consultation and for examination. Three studies [43, 58, 59] examined impacts of EHRs on patients waiting for doctor time. In one study [59], investigators measured the mean patient flow time in a paediatric practice in the USA and found that although the mean patient flow time increased from 56.24 min to 81.43 min one month after the EHR implementation and to 64.60 min 12 months later, patients’ waiting time (check-in to front desk and front desk to triage) actually dropped down by 1.51 and 9.33 min. Their findings suggested the EHR led to more positive results than negative because it reduced waiting for administrative works, allowing more time to be spent on treatment activities. Two studies [54, 56] reported negative impacts of HIS on the waiting time of ED patients. Gray and Fernandes [54] examined the adoption of CPOE in an ED in London Health Sciences Centre with around 100,000 patients per year to determine that CPOE caused an average increase of 5 min in waiting time. A more significant increase in waiting time from 40 to 78 min was observed in a 54,000 patient-per-year ED with the EMR system by Mohan, Bishop, and Mallows [56].

Treatment time is an important component of LOS and it directly influences health outcomes. However, in this review, we could only identify six studies that used this measure to assess the effectiveness of HIS. Unfortunately, these studies did not provide detailed explanation how the HIS affected treatment time. Three studies [22, 23, 44] found that health providers reduced treatment time when using an ED information system and a patient tracking system for their practice. The patient tracking system was used in a paediatric centre with 24,000 visits annually and it reduced the time of faculty paediatricians spent in Exam room from 11.33 to 6.53 min [23]. Meanwhile Baumlin et al. [22] determined a dramatic decrease by 1.90 h in the doctor-to-disposition time after an ED information system was implemented. Two studies about the EHR systems [58, 59] and an asthma management system [60] did not identify a significant difference in treatment caused by the interventions.

TAT is another time-related measure and it was investigated in 6 studies [21, 22, 38, 41, 48, 51]. TAT is defined as the time lapse between when the test is ordered and when the result is available [41]. Four studies [21, 22, 39, 41] examined TAT of the radiology examinations and laboratory results; one study [48] investigated TAT of housekeeping services and one study [51] reported pathology examinations. All of the studies reported impressive reductions in TAT after the implementation of a HIS. For example, Nitrosi et al. [41] noted a decrease in the mean chest exam TAT from 33.9 to 9.62 h.

Finally, boarding time is an important patient flow measure that is often referred to as access block or bed block and it is a main patient flow problem [63]. Two studies [22, 52] examined this measure although Pyron and Carter-Templeton [43] investigated provider discharge-to-nurse discharge time, which can be related to boarding time, but they did not explain or describe how this measure was calculated. Baumlin et al. [22] reported that the use of an ED information system reduced boarding time for the patient by 28% from 6.77 h to 4.90 h. By contrast, Feblowitz et al. [52] noticed an increase in the mean boarding time per patient from 211.2 min to 221.4 min in the long term (1 year after the implementation of an electronic documentation system) in an ED. However, neither study provided a causal relationship between HIS implementation and the changes in boarding time.

In addition to time-related measures, included studies also investigated other important individual outcomes including: four studies on the percentage of patients who left without being seen (LWBS), three studies on patient satisfaction, one each for mortality rate, and readmission rate. LWBS was studied in the ED setting. Three studies [54, 56, 61] reported increases of LWBS percentage with the most significant increase being reported in the study about a CPOE system from 24.3% to 42.0% [54], while Jensen [33] determined a reduction of 7.6%, but this study did not provide any subjective evidence. Patient satisfaction was measured in three studies with one positive result [35], one negative [62], and one neutral result [58]. The EHR system was found to reduce ED patient satisfaction because it increased LOS; however, the negative impacts lasted for only eight weeks before returning to the baseline from before the intervention implementation [62]. One study reported that the use of a patient discharging system [37] was associated with improvement in LOS for early discharge patients without higher rates of readmission. In another study, Inokuchi et al. [32] investigated the impacts of a newly-developed EMR system on the mortality rate at 28 days after hospitalisation and found no changes resulting from the intervention, which is a positive outcome.

Apart from the patient-related outcome measures above, studies also examined organisational outcomes including four studies about hospital costs, and one each for staff satisfaction, film saving and staff stress level. Three studies [33, 47, 53] calculated the reduction in LOS as hospital cost saving. The first study found that EMR systems were associated with 5.9% to 10.3% higher cost per discharge while with the implementation of a patient flow system, Jensen [33] reported that the hospital saved between 67,800 and 214,200 USD. The transition from traditional into digital radiology room through the implementation of a PACS system was found to reduce 90% of the film [41]. Staff satisfaction was examined in a study [49] which reported positive outcomes after the implementation of an electronic prescribing system. In a study about a workflow management system, Li et al. [35] found that the intervention greatly improved sonographers’ productivity while reducing their stress level, which was measured by a 5-point Likert scale. Measures related to organisational outcome are an interesting part of the HIS literature because most of the evidence in patient flow intervention focused primarily on patient-related outcome measures.

#### Impacts on process measures

Studies examined a variety of measures related to staff productivity in clinical processes, and medical guideline adherence. Four studies examined the effect of HIS on the number of medical services performed by the staff. Two studies showed increased number of surgeries [31] and radiology tests [41]. Nitrosi and colleagues [41] studied the impacts of a PACS and found that the number of imaging procedures increased by 7% although the number of technologies and radiologists remained unchanged. An increase of 37% in the number of surgeries after a surgery information system was observed by Gomes and Lapao [31]. However, EHR implementation was found to decrease the number of patients that clinical staff could see [55] although the negative impact was only temporary and resolved three months post-implementation. The implementation of HIS did not change the medical guideline adherence of the staff when they are already providing care that adheres to the relevant guideline [60]. The number of patients seen per shift by medical staff was measured by Mohan, Bishop, and Mallows [56] in an investigation of the effectiveness of an EMR system and the impact was negative. Mathews et al. [37] and Tran et al. [49] both measured the impact of HIS on the percentage of early discharged patients and show positive outcomes. Finally, Tran et al. [49] reported an increase in the number of prescriptions prepared the day before discharge as a positive effect of a prescription system.

#### Impacts on structure measures

Evidence on the impact of HIS on structure measures was more limited than data on process and outcome measures. Six of the 44 studies reported some data on structure measures. These structure measures are related to flow problems facing healthcare organisations and they were studied in ED settings. Almost all of the six studies reported positive impacts of HIS on these structure measures including the number of patient diversions and the number of ED patients with LOS over 12h [33], the proportion of early discharged patients [37], ED avoidance percentage [38], and the number and proportion of access blocks and hospital occupancy rates [27]. The study of Crilly et al. [27] found that the number of access blocks and hospital occupancy rates did not change after the implementation of a patient admission prediction system, but this is actually a positive outcome because the hospital presentations were increasing during the study period. By contrast, in one study, Mohan, Bishop, and Mallows [56] investigated the effect of an EMR system on the percentage of ambulance offloading time of more than 30 min which is also known as ambulance boarding and they found that the percentage went up from 10.5% to 13.3%.

### 5.3 Quality assessment of the included studies

Using the GRADE approach to assess the quality level of the evidence through their study design, two RCT studies [32, 60] were assessed as high quality and 38 observational studies using retrospective or prospective data were rated low quality. Four studies including three expert opinions and one stating improvement without figures did not provide objective evidence and they were rated ‘0’ (the lowest rating). Two studies using multi-method design with both qualitative and quantitative components were rated low quality, based on the assessment of their quantitative component. Details of the quality assessment are provided in S5 File, Quality assessment of the included studies.

## 6. Discussion

### 6.1 Summary of key findings

This systematic review summarised and synthesised evidence from studies about HISs that have been applied to improve patient flow in both inpatient and outpatient settings. Overall, 33 out of the 44 included studies reported positive impacts of HIS on patient flow measures while 7 determined negative impacts, and 4 studies reported no significant impact. Half of the studies focused on patient flow at the departmental level; however, 18 studies reported the impact of HIS on the hospital-wide level and 4 studies reported network-wide impacts on HIS. Healthcare settings adopted at least 17 types of HIS to address patient flow problems and improve care efficiency.

We found that core features of the HIS interventions, that affected patient flow, included patient tracking, documentation management, order entry, patient registration, bed management, decision support, discharge management, prescription management and patient flow reporting. When it comes to the impacts of HIS on specific patient flow measures, most studies focused on outcome measures at both: patient (individual) and organisational level. Changes in individual outcomes were evident in time-related measures including length of stay (LOS), waiting time, treatment time, test turnaround time (TAT), and boarding time, and other measures such as left without being seen and patient satisfaction. Organisational outcome measures were noted in hospital costs, film saving, staff satisfaction, and staff stress level. Process measures and structure measures, although less examined in the included studies than outcome measures, are important measures. While process measures related to staff productivity and guideline adherence, structure measures included flow problems such as patient diversion, access block, hospital occupancy, ambulance offloading time, and ED patient with LOS over 12 h.

Noted HIS benefits included improvements in various patient flow aspects: access to needed information, staff communication, care coordination, work processes, and decision support. Ineffective interaction between hospital units is one of the most common causes of poor patient flow [64]. HISs were effective in fostering care coordination and collaboration among multidisciplinary teams by imposing a common set of flow key performance indicators (KPIs), and metrics into practice. The application of these common, sometimes “simple”, rules help develop common understandings and it is a key to governing complex systems [12]. In addition, the involvement of all team members in the development process of HIS is critical to achieving shared understandings. In this review, the effectiveness of HIS in care coordination was evident in many care processes such as patient check-in [59], elective waiting list management [31], bed management [36, 48], ambulance distribution [38], and discharge [36, 37, 49]. By integrating information from multiple siloed systems, patient flow-related HIS reduce the time needed for care providers to acquire sufficient information to make critical decisions. Real-time data, notifications, and alerts functions are key features that enabled users to get the most updated information in a timely manner. The development of HISs often included redesigning the embedded care process or processes, an opportunity for care settings to eliminate redundant steps and apply best practices to their care processes. Streamlined work processes helped reduce waiting time for test results and free up staff from redundant information [22, 34]. In addition, high degree of automation resulting from the HIS adoption contributed to the reduction in human errors, which can cause medical and health complications, and cognitive workload for hospital staff as they were not required to remember complex rules.

However, it still remains unclear how and why these interventions produced or did not produce positive or negative impacts. Most of the included studies were observational, before and after studies, making it challenging to establish the cause and effect link between HIS interventions and changes in patient flow measures. This has important implications because without a thorough understanding of why and how HIS affected patient flow, it is difficult to generalise the findings to other healthcare settings.

### 6.2 Strength and limitations

To date, several systematic reviews have been conducted to investigate interventions addressing patient flow problems; however, they focused mostly on operational methods such as triage, fast track, streaming [2]. Systematic reviews on the impact of HIS on patient flow are small in number and limited to single specific systems such as CPOE [5]. To the best of our knowledge, this review was the first attempt to evaluate a broad range of HISs applied in patient flow management. The novelty of this review lies in its research aim, and inclusion criteria, unlike most previous reviews on patient flow interventions, here, we included different types of HISs and broad scope of healthcare settings including departmental, organisational and network levels. Our findings provide different stakeholders with important insights for their implementation and adoption of HISs to optimise patient flow.

However, this review has several limitations. The first relates to the heterogeneous nature of the search terminology and the quantity and scope of the evidence. Although we conducted a comprehensive search, in many important domains, we could only identify a limited number of studies. The second limitation relates to the synthesis of varied outcomes and a broad range of HISs. In this review, we attempted to address this limitation by adopting analytic frameworks, which were based on domain experts and published literature, and by synthesising not only the health information system but also their functional features. Third, descriptions of the HIS interventions and the implementation process were often very limited, making it challenging to fully assess the system features and associated benefits. Fourth, most of the included studies are before-and-after, observational studies and therefore understanding of how and why HISs affected patient flow outcomes was very limited. Finally, we decided not to include a meta-analysis because of the diverse, heterogeneous outcomes reported in the included studies. A meta-analysis, in this case, is inappropriate and can be more of a hindrance than a help [65].

### 6.3 Implications for patient flow management practice

Hospitals and care centres have implemented several interventions to tackle patient flow problems to deliver optimal care. However, up until recently, most of the efforts were focused on addressing ED overcrowding problems [3, 66, 67]. It is evident in the literature that focusing solely on ED problems will not likely achieve optimal flow because EDs do not operate separately, rather they are part of an interconnected system [68]. Therefore, literature has urged that patient flow needs to be viewed from the whole system of care viewpoint and called for a shift from ED-focused to system-wide or hospital-wide interventions [12, 69]. However, the gap between understanding the problem and having solutions to solve the problem seems still far. For example, even a holistic approach like Lean healthcare was still attached to a specific department or care process [4]. The frequently reported intervention to improve inpatient flow was implementing a specialised staff or team to coordinate patient flow across hospital units; however, the solution still posed significant challenges [3]. This systematic review found that apart from 22 studies focusing on department level, many studies reported hospital-wide or even network-wide level. HISs’ potential to address patient flow at the hospital-wide level were noted in their ability to improve communication between multidisciplinary teams [25, 36], enhance care coordination [36, 49], improve access to needed information [41, 43], and streamline care processes [25, 59]. One of the prominent causes of admission bottleneck is inefficient discharges [68] because any delays in inpatient discharge will increase hospital occupancy and ED overcrowding [69]. HISs showed their effectiveness in discharge prediction and established standardised discharge criteria for improving the discharge process [37]. These “medical-readiness criteria” have been shown to facilitate efficient planning and care coordination [37]. Addressing patient flow problems sometimes goes beyond hospital scope to a higher level of network-wide scope. A dashboard system was developed in Alberta, Canada to address ED overcrowding by coordinating emergency services between different emergency rooms within the region [9]. HISs were also used within a network of different hospitals to address the need for rehabilitation care services and improve the consultation process [42]. HISs can be scalable to a nationwide level to reduce waiting time for elective surgical patients [31]. By providing information about capacity, occupancy and demand, they can be highly effective in addressing the mismatch between supply and demand to improve patient flow.

### 6.4 Implications for future research

Moving forward, this review suggests important areas for future research in the field. First, additional studies need to explore barriers and facilitators of the HISs related to patient flow management. This will offer valuable implications for healthcare organisations to drive their HIS project to success and derive the most from their investment. Second, learning about the effectiveness of HISs on patient flow and associated factor during the post-implementation phase could help to advance the field. This is because of the evolutionary nature of HIS development in which factors associated with the application of HIS can be captured and used as lessons learned for the next evolution of the HIS [70]. In this review, only the study of Inokuchi et al. [32] addressed this topic. Patient flow is often negatively affected during the implementation of HIS because of changes in the workflow and human resources. Although the effect seemed temporary, learning about these periods and associated factors will bring implications for researchers and policymakers when considering the project timeline and expected challenges. Furthermore, although HISs are found to help healthcare organisations address patient flow management areas such as care coordination, timely access to information, and communication barriers, understanding why and how HIS could enhance each of these aspects can be extremely helpful. Part of the reasons to explain this is because most of the selected studies in this review did not include adequate details of the underlining technologies of the HIS interventions such as: what are the technical supports and architectures, what are the input and output data, or how the output data are represented in the user interface. The lack of technical specifications of the HIS interventions made it hard to fully comprehend how they contributed to the changes in patient flow management. Finally, during the last two years, the COVID-19 pandemic has completely disrupted patient flow management all over the world. Yet, we could not identify any studies on the role of HIS in remedying the impacts of the pandemic on patient flow.

## 7. Conclusion

Health information systems (HISs) provide clear benefits in managing patient flow over traditional paper record management systems. However, without a systematic evaluation and summary of the available evidence, stakeholders interested in adopting HISs in healthcare settings for patient low management might be lost in the ocean of information. This is especially true when it comes to the questions of what HIS to invest, what benefits and impacts to expect and how to maximize the values from their investment. This systematic review has revealed an increasing interest in adopting HIS to address patient flow issues in healthcare settings in the last decade. HISs can be effective solutions for patient flow management at the organisational-wide or even network-wide levels due to their great scalability and integrability. HISs were often found to be effective in improving communication and care coordination between team members, providing timely access to high quality information for decision making, and streamlining care processes. These improvements contributed to more efficient patient flow throughout the care continuum. As more healthcare and health-related data are generated, there are great opportunities for HISs such as decision support systems, and dashboard systems to help healthcare organisations harness the power of big-data analytics and achieve optimal patient flow. This review shows that HISs can impact various aspects of patient flow at different levels of care; however, how and why they delivered the impacts will require further research.

## Supporting information

S1 File Search strategy. (DOCX)

S2 File Reported benefits of HISs on patient flow management. (DOCX)

S3 File Table of all included studies and findings. (XLSX)

S4 File Example of data extraction form. (XLSX)

S5 File Quality assessment of the included studies. (DOCX)

S6 File PRISMA checklist. (DOC)

## References

[1] Gualandi R, Masella C, Viglione D, Tartaglini D. Exploring the hospital patient journey: What does the patient experience? PloS one. 2019 Dec 5;14(12): e0224899. doi: 10.1371/journal.pone.0224899 . 31805061 PMC6894823

[2] De Freitas L, Goodacre S, O’Hara R, Thokala P, Hariharan S. Interventions to improve patient flow in emergency departments: an umbrella review. Emergency Medicine Journal. 2018 Oct 1;35(10):626–37. doi: 10.1136/emermed-2017-207263 . 30093379

[3] Winasti W, Elkhuizen S, Berrevoets L, van Merode G, Berden H. Inpatient flow management: a systematic review. International journal of health care quality assurance. 2018 Aug 13. doi: 10.1108/IJHCQA-03-2017-0054 . 30354877

[4] Tlapa D, Zepeda-Lugo CA, Tortorella GL, Baez-Lopez YA, Limon-Romero J, Alvarado-Iniesta A, et al. Effects of lean healthcare on patient flow: a systematic review. Value in Health. 2020 Feb 1;23(2):260–73. doi: 10.1016/j.jval.2019.11.002 32113632

[5] Georgiou A, Prgomet M, Paoloni R, Creswick N, Hordern A, Walter S, et al. The effect of computerized provider order entry systems on clinical care and work processes in emergency departments: a systematic review of the quantitative literature. Annals of emergency medicine. 2013 Jun 1;61(6):644–53. doi: 10.1016/j.annemergmed.2013.01.028 23548404

[6] Mohiuddin S, Busby J, Savović J, Richards A, Northstone K, Hollingworth W, et al. Patient flow within UK emergency departments: a systematic review of the use of computer simulation modelling methods. BMJ open. 2017 May 1;7(5): e015007. doi: 10.1136/bmjopen-2016-015007 28487459 PMC5566625

[7] Siddique SM, Tipton K, Leas B, Greysen SR, Mull NK, Lane-Fall M, et al. Interventions to Reduce Hospital Length of Stay in High-risk Populations: A Systematic Review. JAMA network open. 2021 Sep 1;4(9): e2125846–e2125846. doi: 10.1001/jamanetworkopen.2021.25846 34542615 PMC8453321

[8] Trotzky D, Posner L, Mosery J, Cohen A, Avisar S, Pachys G. Do automatic push notifications improve patient flow in the emergency department? analysis of an ED in a large medical center in Israel. Plos one. 2021 Oct 7;16(10): e0258169. doi: 10.1371/journal.pone.0258169 34618849 PMC8496857

[9] McLeod B, Zaver F, Avery C, Martin DP, Wang D, Jessen K, et al. Matching capacity to demand: a regional dashboard reduces ambulance avoidance and improves accessibility of receiving hospitals. Academic Emergency Medicine. 2010 Dec;17(12):1383–9. doi: 10.1111/j.1553-2712.2010.00928.x 21122023

[10] Hong WS, Haimovich AD, Taylor RA. Predicting hospital admission at emergency department triage using machine learning. PloS one. 2018 Jul 20;13(7): e0201016. doi: 10.1371/journal.pone.0201016 30028888 PMC6054406

[11] Blaya JA, Shin SS, Yagui M, Contreras C, Cegielski P, Yale G, et al. Reducing communication delays and improving quality of care with a tuberculosis laboratory information system in resource poor environments: a cluster randomized controlled trial. PloS one. 2014 Apr 10;9(4): e90110. doi: 10.1371/journal.pone.0090110 24721980 PMC3982951

[12] Rutherford PA, Provost LP, Kotagal UR, Luther K, Anderson A. Achieving hospital-wide patient flow. IHI White Paper. Cambridge: Institute for Healthcare Improvement. 2017.

[13] Resar R, Nolan K, Kaczynski D, Jensen K. Using real-time demand capacity management to improve hospital wide patient flow. The Joint Commission Journal on Quality and Patient Safety. 2011 May 1;37(5):217–AP3. doi: 10.1016/s1553-7250(11)37029-8 21618898

[14] Bergs J, Vandijck D, Hoogmartens O, Heerinckx P, Van Sassenbroeck D, Depaire B, et al. Emergency department crowding: time to shift the paradigm from predicting and controlling to analysing and managing. International Emergency Nursing. 2016 Jan 1; 24:74–7. doi: 10.1016/j.ienj.2015.05.004 27170954

[15] Wohlin C. Guidelines for snowballing in systematic literature studies and a replication in software engineering. In Proceedings of the 18th international conference on evaluation and assessment in software engineering. 2014 May 13 (pp. 1–10). 10.1145/2601248.2601268.

[16] Moher D, Liberati A, Tetzlaff J, Altman DG, PRISMA Group*. Preferred reporting items for systematic reviews and meta-analyses: the PRISMA statement. Annals of internal medicine. 2009 Aug 18;151(4):264–9. doi: 10.1371/journal.pmed.1000097 19622511

[17] Covidence systematic review software, Veritas Health Innovation, Melbourne, Australia. Available at www.covidence.org.

[18] Schünemann H, Brożek J, Guyatt G, Oxman A. GRADE handbook for grading quality of evidence and strength of recommendations. Updated October 2013. The GRADE Working Group, 2013.

[19] Donabedian A. The quality of care: how can it be assessed? Jama. 1988 Sep 23;260(12):1743–8. doi: 10.1001/jama.260.12.1743 3045356

[20] Alamo ST, Wagner GJ, Sunday P, Wanyenze RK, Ouma J, Kamya M, et al. Electronic medical records and same day patient tracing improves clinic efficiency and adherence to appointments in a community based HIV/AIDS care program, in Uganda. AIDS and Behaviour. 2012 Feb;16(2):368–74. doi: 10.1007/s10461-011-9996-9 21739285 PMC3872059

[21] Arenson R. Why bother with a computerized scheduling system? Journal of Digital Imaging. 1988 Nov;1(1):24–7. doi: 10.1007/BF03167747 3154654

[22] Baumlin KM, Shapiro JS, Weiner C, Gottlieb B, Chawla N, Richardson LD. Clinical information system and process redesign improves emergency department efficiency. The Joint Commission Journal on Quality and Patient Safety. 2010 Apr 1;36(4):179–AP1. doi: 10.1016/s1553-7250(10)36030-2 20402375

[23] Borowitz SM. Impact of a computerized patient tracking system in a pediatric clinic. In Proceedings of the AMIA Annual Fall Symposium 1996 (p. 508). American Medical Informatics Association. 8947718 PMC2233189

[24] Cho SJ, Jeong J, Han S, Yeom S, Park SW, Kim HH, et al. Decreased emergency department length of stay by application of a computerized consultation management system. Academic Emergency Medicine. 2011 Apr;18(4):398–402. doi: 10.1111/j.1553-2712.2011.01039.x 21496143

[25] Clark KW, Moller S, O’Brien L. Electronic patient journey boards a vital piece of the puzzle in patient flow. Australian Health Review. 2014 May 12;38(3):259–64. doi: 10.1071/AH13192 24814229

[26] Clark KW, Whiting E, Rowland J, Thompson LE, Missenden I, Schellein G. Breaking the mould without breaking the system: the development and pilot of a clinical dashboard at The Prince Charles Hospital. Australian Health Review. 2013 May 24;37(3):304–8. doi: 10.1071/AH12018 23701819

[27] Crilly JL, Boyle J, Jessup M, Wallis M, Lind J, Green D, et al. The implementation and evaluation of the patient admission prediction tool: assessing its impact on decision-making strategies and patient flow outcomes in 2 Australian hospitals. Quality management in health care. 2015 Oct 1;24(4):169–76. doi: 10.1097/QMH.0000000000000070 26426317

[28] Dackiewicz D, Bergsneider C, Piraino D. Impact of digital radiography on clinical workflow and patient satisfaction. Journal of Digital Imaging. 2000 May;13(1):200–1. doi: 10.1007/BF03167662 10847400 PMC3453299

[29] Daniel GW, Ewen E, Willey VJ, Reese IV CL, Shirazi F, et al. Efficiency and economic benefits of a payer‐based electronic health record in an emergency department. Academic Emergency Medicine. 2010 Aug;17(8):824–33. doi: 10.1111/j.1553-2712.2010.00816.x 20670319

[30] Ewing A, Rogus J, Chintagunta P, Kraus L, Sabol M, Kang H. A systems approach to improving patient flow at UVA Cancer Center using Real-Time Locating System. In2017 Systems and Information Engineering Design Symposium (SIEDS) 2017 Apr 28 (pp. 259–264). IEEE. 10.1109/SIEDS.2017.7937727

[31] Gomes P, Lapão LV. The SIGLIC system for improving the access to surgery in Portugal. Electronic Journal of Information Systems Evaluation. 2011 Jan 1;14(1): pp160–166.

[32] Inokuchi R, Sato H, Iwagami M, Komaru Y, Iwai S, Gunshin M, et al. Impact of a new medical record system for emergency departments designed to accelerate clinical documentation: a crossover study. Medicine. 2015 Jul;94(26). doi: 10.1097/MD.0000000000000856 26131837 PMC4504572

[33] Jensen J. United hospital increases capacity usage, efficiency with patient-flow management system. Journal of healthcare information management: JHIM. 2004 Jan 1;18(3):26–31. 15301414

[34] Jung KY, Kim S, Kim K, Lee EJ, Kim K, Lee J, et al. Frequent mobile electronic medical records users respond more quickly to emergency department consultation requests: Retrospective Quantitative Study. JMIR mHealth and uHealth. 2020 Feb 14;8(2): e14487. doi: 10.2196/14487 32130157 PMC7055754

[35] Li MF, Tsai JC, Chen WJ, Lin HS, Pan HB, Yang TL. Redefining the sonography workflow through the application of a departmental computerized workflow management system. International Journal of Medical Informatics. 2013 Mar 1;82(3):168–76. doi: 10.1016/j.ijmedinf.2012.06.001 22762863

[36] Maloney CG, Wolfe D, Gesteland PH, Hales JW, Nkoy FL. A tool for improving patient discharge process and hospital communication practices: The Patient Tracker. In AMIA Annual Symposium Proceedings 2007 (Vol. 2007, p. 493). American Medical Informatics Association. 18693885 PMC2655791

[37] Mathews KS, Corso P, Bacon S, Jenq GY. Using the red/yellow/green discharge tool to improve the timeliness of hospital discharges. The Joint Commission Journal on Quality and Patient Safety. 2014 Jun 1;40(6):243–AP3. doi: 10.1016/s1553-7250(14)40033-3 25016672 PMC4419567

[38] Mekhjian HS, Kumar RR, Kuehn L, Bentley TD, Teater P, Thomas A, et al. Immediate benefits realized following implementation of physician order entry at an academic medical center. Journal of the American Medical Informatics Association. 2002 Sep 1;9(5):529–39. doi: 10.1197/jamia.m1038 12223505 PMC346640

[39] Nazar H, Brice S, Akhter N, Kasim A, Gunning A, Slight SP, et al. New transfer of care initiative of electronic referral from hospital to community pharmacy in England: a formative service evaluation. BMJ open. 2016 Oct 1;6(10): e012532. doi: 10.1136/bmjopen-2016-012532 27742628 PMC5073802

[40] Nelson P, Bell AJ, Nathanson L, Sanchez LD, Fisher J, Anderson PD. Ethnographic analysis on the use of the electronic medical record for clinical handoff. Internal and Emergency Medicine. 2017 Dec;12(8):1265–72. doi: 10.1007/s11739-016-1567-7 27832465

[41] Nitrosi A, Borasi G, Nicoli F, Modigliani G, Botti A, Bertolini M, et al. A filmless radiology department in a full digital regional hospital: quantitative evaluation of the increased quality and efficiency. Journal of digital imaging. 2007 Jun;20(2):140–8. doi: 10.1007/s10278-007-9006-y 17318704 PMC1896266

[42] Poulos CJ, Gazibarich BM, Eagar K. Supporting work practices, improving patient flow and monitoring performance using a clinical information management system. Australian Health Review. 2007;31(5):79–85. doi: 10.1071/ah070s79 17402909

[43] Pyron L, Carter-Templeton H. Improved Patient Flow and Provider Efficiency After the Implementation of an Electronic Health Record. CIN: Computers, Informatics, Nursing. 2019 Oct 1;37(10):513–21. doi: 10.1097/CIN.0000000000000553 31385814

[44] Risko N, Anderson D, Golden B, Wasil E, Barrueto F, Pimentel L, et al. The impact of electronic health record implementation on emergency physician efficiency and patient throughput. In Healthcare 2014 Sep 1 (Vol. 2, No. 3, pp. 201–204). Elsevier. doi: 10.1016/j.hjdsi.2014.06.003 . 26250507

[45] Rolls D, Khanna S, Lloyd N, Reeson A, Jayasena R, McCormick C, et al. Before-after evaluation of patient length of stay in a rehabilitation context following implementation of an electronic patient journey board. International Journal of Medical Informatics. 2020 Feb 1; 134:104042. doi: 10.1016/j.ijmedinf.2019.104042 31855847

[46] Sicotte C, Lapointe J, Clavel S, Fortin MA. Benefits of improving processes in cancer care with a care pathway-based electronic medical record. Practical radiation oncology. 2016 Jan 1;6(1):26–33. doi: 10.1016/j.prro.2015.08.011 26598908

[47] Stone WM, Smith BE, Shaft JD, Nelson RD, Money SR. Impact of a computerized physician order-entry system. Journal of the American College of Surgeons. 2009 May 1;208(5):960–7. doi: 10.1016/j.jamcollsurg.2009.01.042 19476871

[48] Tortorella F, Ukanowicz D, Douglas-Ntagha P, Ray R, Triller M. Improving bed turnover time with a bed management system. The Journal of Nursing Administration. 2013 Jan 1;43(1):37–43. doi: 10.1097/NNA.0b013e3182785fe7 23232178

[49] Tran T, Hardidge A, Heland M, Taylor SE, Garrett K, Mitri E, et al. Slick scripts: impact on patient flow targets of pharmacists preparing discharge prescriptions in a hospital with an electronic prescribing system. Journal of Evaluation in Clinical Practice. 2017 Apr;23(2):333–9. doi: 10.1111/jep.12615 27524695

[50] Welch SJ, Jones SS, Allen T. Mapping the 24-hour emergency department cycle to improve patient flow. The Joint Commission Journal on Quality and Patient Safety. 2007 May 1;33(5):247–55. doi: 10.1016/s1553-7250(07)33029-8 17503680

[51] Westbrook JI, Georgiou A, Lam M. Does computerised provider order entry reduce test turnaround times? A before-and-after study at four hospitals. In Medical Informatics in a United and Healthy Europe 2009 (pp. 527–531). IOS Press. 10.3233/978-1-60750-044-5-527 19745367

[52] Feblowitz J, Takhar SS, Ward MJ, Ribeira R, Landman AB. A custom-developed emergency department provider electronic documentation system reduces operational efficiency. Annals of emergency medicine. 2017 Nov 1;70(5):674–82. doi: 10.1016/j.annemergmed.2017.05.032 28712608 PMC5653416

[53] Furukawa MF, Raghu TS, Shao BB. Electronic medical records, nurse staffing, and nurse‐sensitive patient outcomes: Evidence from California hospitals, 1998–2007. Health services research. 2010 Aug;45(4):941–62. doi: 10.1111/j.1475-6773.2010.01110.x 20403065 PMC2910563

[54] Gray A, Fernandes CM, Van Aarsen K, Columbus M. The impact of computerized provider order entry on emergency department flow. Canadian Journal of Emergency Medicine. 2016 Jul;18(4):264–9. doi: 10.1017/cem.2016.11 27018711

[55] Kennebeck SS, Timm N, Farrell MK, Spooner SA. Impact of electronic health record implementation on patient flow metrics in a pediatric emergency department. Journal of the American Medical Informatics Association. 2012 May 1;19(3):443–7. doi: 10.1136/amiajnl-2011-000462 22052897 PMC3341791

[56] Mohan MK, Bishop RO, Mallows JL. Effect of an electronic medical record information system on emergency department performance. Medical journal of Australia. 2013 Mar;198(4):201–4. doi: 10.5694/mja12.10499 23451963

[57] Vartak S, Crandall DK, Brokel JM, Wakefield DS, Ward MM. Professional practice and innovation: transformation of emergency department processes of care with EHR, CPOE, and ER event tracking systems. Health Information Management Journal. 2009 Jun;38(2):27–32. 10.1177/183335830903800204 19546485

[58] Ward MJ, Landman AB, Case K, Berthelot J, Pilgrim RL, Pines JM. The effect of electronic health record implementation on community emergency department operational measures of performance. Annals of emergency medicine. 2014 Jun 1;63(6):723–30. https://doi.org/10.1016%2Fj.annemergmed.2013.12.019 10.1016/j.annemergmed.2013.12.019 PMC4059411 24412667

[59] Bushelle-Edghill J, Brown JL, Dong S. An examination of EHR implementation impacts on patient-flow. Health Policy and Technology. 2017 Mar 1;6(1):114–20. 10.1016/j.hlpt.2016.11.005.

[60] Dexheimer JW, Abramo TJ, Arnold DH, Johnson K, Shyr Y, Ye F, et al. Implementation and evaluation of an integrated computerized asthma management system in a pediatric emergency department: a randomized clinical trial. International journal of medical informatics. 2014 Nov 1;83(11):805–13. doi: 10.1016/j.ijmedinf.2014.07.008 25174321 PMC5460074

[61] Tall JM, Hurd M, Gifford T. Minimal impact of an electronic medical records system. The American journal of emergency medicine. 2015 May 1;33(5):663–6. doi: 10.1016/j.ajem.2015.02.022 25744149

[62] Ward MJ, Froehle CM, Hart KW, Collins SP, Lindsell CJ. Transient and sustained changes in operational performance, patient evaluation, and medication administration during electronic health record implementation in the emergency department. Annals of emergency medicine. 2014 Mar 1;63(3):320–8. doi: 10.1016/j.annemergmed.2013.08.019 24041783 PMC3961764

[63] Pines JM, Hilton JA, Weber EJ, Alkemade AJ, Al Shabanah H, Anderson PD, et al. International perspectives on emergency department crowding. Academic Emergency Medicine. 2011 Dec;18(12):1358–70. doi: 10.1111/j.1553-2712.2011.01235.x 22168200

[64] White CM, Statile AM, White DL, Elkeeb D, Tucker K, Herzog D, et al. Using quality improvement to optimise paediatric discharge efficiency. BMJ quality & safety. 2014 May 1;23(5):428–36. doi: 10.1136/bmjqs-2013-002556 24470173

[65] Higgins JP, Thomas J, Chandler J, Cumpston M, Li T, Page MJ, et al. Cochrane handbook for systematic reviews of interventions. John Wiley & Sons; 2019 Sep 23. 10.1002/9781119536604. PMC10284251 31643080

[66] Eitel DR, Rudkin SE, Malvehy MA, Killeen JP, Pines JM. Improving service quality by understanding emergency department flow: a White Paper and position statement prepared for the American Academy of Emergency Medicine. The Journal of emergency medicine. 2010 Jan 1;38(1):70–9. doi: 10.1016/j.jemermed.2008.03.038 18514465

[67] Oredsson S, Jonsson H, Rognes J, Lind L, Göransson KE, Ehrenberg A, et al. A systematic review of triage-related interventions to improve patient flow in emergency departments. Scandinavian journal of trauma, resuscitation and emergency medicine. 2011 Dec;19(1):1–9. doi: 10.1186/1757-7241-19-43 21771339 PMC3152510

[68] Haraden C, Resar R. Patient flow in hospitals: understanding and controlling it better. Frontiers of health services management. 2004 Jul 1;20(4):3. 15219146

[69] Institute for Healthcare Improvement. Optimizing patient flow: moving patients smoothly through acute care settings. Innovation Series. 2003:1–1.

[70] Nguyen Q, Meredith R, Burstein F. A Comparative Study of Critical Success Factors for General and Healthcare Business Intelligence Systems. 29th Australasian Conference on Information Systems, ACIS 2018. 2018, December 03–05; Sydney, Australia. UTS ePRESS, 12 p. 4. 10.5130/acis2018.cb.

