# Supplementary material for: Understanding the impacts of health information systems on patient flow management: A systematic review across several decades of research
Source: PLoS One. 2022 Sep 12;17(9):e0274493. doi: 10.1371/journal.pone.0274493 (PMC9467348; doi:10.1371/journal.pone.0274493)
Supplement: S1 File — (DOCX) [file pone.0274493.s001.docx]

S1 File:

**Medline via Ovid search strategy:**

| 1. | exp Information Systems/ |
| --- | --- |
| 2. | exp Medical Records Systems, Computerized/ |
| 3. | EMR.mp. |
| 4. | EHR.mp. |
| 5. | "Decision support system*".mp. |
| 6. | DSS.mp. |
| 7. | "Business intelligence system*".mp. |
| 8. | exp Hospital Information Systems/ |
| 9. | "BI system*".mp. |
| 10. | *Medical Order Entry Systems/ |
| 11. | "bed board".mp. |
| 12. | 1 or 2 or 3 or 4 or 5 or 6 or 7 or 8 or 9 or 10 or 11 |
| 13. | exp Crowding/ or exp "Length of Stay"/ |
| 14. | "patient flow".mp. |
| 15. | "hospital flow".mp. |
| 16. | overcrowding.mp. |
| 17. | "access block*".mp. |
| 18. | *Bed Occupancy/ |
| 19. | "waiting time".mp. |
| 20. | 13 or 14 or 15 or 16 or 17 or 18 or 19 |
| 21. | 12 and 20 |
| 22. | limit 21 to (English language) |

**Embase via Ovid search strategy**

| 1. | exp medical information system/ or *information system/ |
| --- | --- |
| 2. | exp hospital information system/ |
| 3. | exp electronic health record/ |
| 4. | exp electronic medical record/ |
| 5. | *decision support system/ or *clinical decision support system/ |
| 6. | DSS.mp. |
| 7. | "business intelligence system*".mp. |
| 8. | "BI system*".mp. |
| 9. | exp computerized provider order entry/ |
| 10. | "bed board".mp. |
| 11. | *"length of stay"/ |
| 12. | "patient flow*".mp. |
| 13. | "hospital flow*".mp. |
| 14. | crowding.mp. |
| 15. | "access block*".mp. |
| 16. | "waiting time".mp. |
| 17. | "patient throughput".mp. |
| 18. | "patient journey".mp. |
| 19. | 1 or 2 or 3 or 4 or 5 or 6 or 7 or 9 or 10 |
| 20. | overcrowding.mp. |
| 21. | 11 or 12 or 13 or 14 or 15 or 16 or 17 or 18 or 20 |
| 22. | 19 and 21 |
| 23. | 19 and 21 |

**ACM Library Search Strategy**

query: { AllField:("information system*" OR "electronic health record*" OR "EHR" OR "Electronic medical record*" OR "decision support system*" OR DSS OR "Business intelligence system*" OR "Order entry system*" OR "Bed board") AND AllField:("patient flow*" OR "hospital flow*" OR "patient journey*" OR "patient throughput" OR "overcrowding" OR "Crowding" OR "waiting time" OR "access block*" OR "length of stay" ) }

**CINAHL Search Strategy**

(((MM "Information Systems") OR (MM "Data Warehouse") OR (MH "Health Information Systems+") OR (MM "Image Retrieval Systems") OR (MM "Integrated Advanced Information Management Systems") OR (MM "Management Information Systems")) OR ((MH "Electronic Health Records+") OR (MH "Patient Record Systems+")) OR ehr OR ((MM "Electronic Order Entry")) OR ("electronic medical record*") OR emr OR ((MM "Decision Support Systems, Clinical") OR (MH "Clinical Information Systems+") OR (MM "Emergency Service Information Systems") OR (MM "Health Information Networks") OR (MM "Home Health Care Information Systems") OR (MM "Hospital Information Systems") OR (MH "Nursing Information Systems+")) OR DSS OR ("business intelligence system*") OR ("BI system*") OR (electronic bed board)) AND (("patient flow*") OR ("patient throughput") OR ("patient journey") OR ((MM "Crowding")) OR overcrowding OR ("access block*") OR ("waiting time") OR ((MM "Length of Stay")))
