# Supplementary material for: Understanding the impacts of health information systems on patient flow management: A systematic review across several decades of research
Source: PLoS One. 2022 Sep 12;17(9):e0274493. doi: 10.1371/journal.pone.0274493 (PMC9467348; doi:10.1371/journal.pone.0274493)
Supplement: S2 File — (DOCX) [file pone.0274493.s002.docx]

**S2 File: Reported benefits of HIS on patient flow management**

| Benefits | Details |
| --- | --- |
| Automated manual tasks | - High automation degree delivered by the system reduced human errors in patient identification and examination scheduling. The staff are not required to remember complex scheduling rules [21]. - The system saved the time of manual work by automatically sorting patient into suitable examination rooms [35]. |
| Facilitated care coordination | - The system improved coordination efficiency of the check-in to triage processes in the front office [59]. - Allowed many hospitals to boost the participant surgeons and the efficiency of using operating rooms [31]. - Facilitated multidisciplinary teamwork and cooperation between staff of different fields and background to achieve a common goal [35]. - Improved coordination and communication between disciplines to establish more efficient process of managing beds [36]. - Streamlined ambulance distribution and emergency medical service coordination between regional hospitals to reduce ambulance diversion hours and offload times [38]. - Assisted the staff in other discharge coordination activities by providing information about how long the medications would take to organise [49]. |
| Staff productivity | - Provided a decent productivity indicator by showing the number of patients per day, per provider and per urgent care [43]. |
| Supported decision making | - Allowed faster referral-to-treatment times and quicker decision making by team members by offering real time data [25]. - Is now an integral part of bed management decision making in 31 public hospitals in Queensland, Australia [27]. - Provided physicians faster access to more complete and detailed information of patient’s history to engage in more analytical decision-making [29]. - A newly added function allowed staff to attach and view physical findings to primary complaints for improving staff satisfaction and patient safety [32]. - Reduced decision making time for ED clinicians by timely delivery of test results and alerting staff then test results were available, translating into less wait times, faster treatment and patient throughput [32]. |
| Improved work processes | - The rapid patient identification feature was introduced into the redesign of the registration process and this reduced door-to-door time. Streamlined the laboratory and radiology ordering process eliminated waiting for old paper records and reducing test turnaround time [22]. - Removed the front desk ledge to boost the efficiency of the sign-in check-in process [59]. - Eliminated redundant communication, which used to be done through text message and phone calls, and delivered patient information to the users’ mobile EMR [34]. - Eliminated typing of off-line reports and freed up staff for other tasks. Delivered images and reports as soon as they are available to allow early treatment and safe discharge of patients [34]. - Added quick-registration feature to the registration process to remove waiting for full registration procedures, streamline the process and reduce length of stay [57]. - Allowed nurses to spend more time with patients by streamlining patient flow management [33]. |
| Improved communication between staff and between staff and patients | - Better communication between staff created by the patient tracking system resulted in significant reduction in waiting time for patients [23]. - Improved communication between team members saved time and increased efficiency [25]. - Removed communication barriers between staff through the system implementation principles [31]. - Physicians, consultants and nurses used Patient Tracker System to communicate with each other and get updates of patients’ discharge throughout the day [36]. - The eSignout codified formal interactions between ED staff and improved interdisciplinary relationship. Decreased communication errors and omissions [40]. - Provided hospital staff with instant access to patients’ status so that they can update waiting families to help them release frustration [33]. |
| Improved access to needed information | - Provided instantaneous access to history ED charts and hospital data from outside the ED. Allowed clinicians to obtain information faster, stay apprised of changes in patient’s conditions and prevent duplicate diagnostic studies [22]. - Displayed relevant clinical information in a timely manner to facilitate strong clinician engagement and acceptance [25]. - Overcome a previous problem of missing information during the patient handoffs process by providing complete set of information to improve efficiency and safety [40]. - Provided immediate access to necessary images and reports for the staff to begin early treatment and safely discharge patients [41]. - Provided real-time information about the date of referral and patient’s location by hospital and ward to rehabilitation medical consultants and registrars. Prevented duplicate data entry [42]. - Emergency Room event tracking alerted staff when the test results are ready to reduce the waiting time [57]. |
| Best practice application | - The system entailed the implementation of known process redesign principles, methods, and changes to the current practice that led to the desired and unanticipated benefits [25]. - Evidence-based care and adherence to performance indicators are facilitated through the imbedded service order sets and system templates [57]. |
| Improved trust and relationship between staff | - Ensured content authentication and enhanced trust among staff [40]. |
| Assisted resource allocation or demand management | - Created a balance between demand and supply by optimizing dispatch through taking in account various aspects related to patient load in the hospital and prehospital environments [38]. - Allowed bed managers to view demand for rehabilitation beds across all rehabilitation facilities to balance demand for beds across different sites [42]. - Identified patient arrival patterns to help the hospital to anticipate the resources and services required for efficient patient flow. Mapped out the link between severity and resource consumption [50]. |
| Integrated information from siloed systems | - Provided immediate access to history ED charts and hospital data from outside of the ED [22]. - The integration of emergency department information with other hospital systems removed the need to log on to different systems for necessary radiology data and the associated delays [23]. - The integration with Radiology Information system reduced the times patients spent in the department [28]. |
| Improved patient satisfaction | - Improved patient experience by increasing their time spent with the service provider and minimizing the waiting time [59]. - Patient satisfaction has been enhanced because the workflow management system has reduced exam waiting time and informing when the next exams will occur through the real-time monitoring function [35]. - Allowed the staff to better control of their workday and improved staff satisfaction and retention [33]. |
| Improved staff satisfaction | - Improved physicians’ satisfaction by reducing the times for clinical documentation and allowing users to enter negative findings and abnormalities in examination results [32]. - Relieved sonographers’ stress level by providing a bigger picture of the examination room and creating a dynamic work environment [35]. |
| Improved staff’s awareness | - Heightened the staff’s awareness of overall patient flow which leads to reduction in average time staff spend in examination rooms [23]. - Displayed patients’ progress to the planned discharge date so that all team members are aware ‘at a glance’ when patients are due for discharge and create plan accordingly [25]. - Allowed sonographers to understand the waiting condition in the examination room which is opposite to the complete ignorant prior to the intervention [35]. |
| Bed control | - Patient flow is better controlled through advanced scheduling. Efficiency is improved and average examination time is reduced [21]. - Patient admission prediction system can be beneficial in informing bed management strategies [27]. - Patient Tracker System helped the staff to identify currently available beds and beds will be available for the next several hours [26]. |
| Assisted discharge assessment and planning | - Allowed care managers to identify patients with the need for home care arrangement or other discharge planning assistance [36]. |
| Bottleneck identification | - The newly developed data tables of “patient locations” and “daily patient experience” help identify flow problems that are otherwise challenging to detect and analyse how patient move through a multi-clinic facility over time [30]. |
| Information standardisation | - Validated information consistency and rejected non-compliant data to ensure the quality of information [31]. - Standardised the quality of transfer information and solidified communication [40]. |
