# Supplementary material for: Understanding the impacts of health information systems on patient flow management: A systematic review across several decades of research
Source: PLoS One. 2022 Sep 12;17(9):e0274493. doi: 10.1371/journal.pone.0274493 (PMC9467348; doi:10.1371/journal.pone.0274493)
Supplement: S5 File — (DOCX) [file pone.0274493.s005.docx]

# **S5: Quality assessment of the included studies**

| First Author, Year, Country | HIS | Study Design | GRADE Level of Evidence | Notes |
| --- | --- | --- | --- | --- |
| Dexheimer, 2014, USA | Asthma Management System | Prospective RCT | high |  |
| Tortorella, 2013, USA | Bed Management System | Expert opinion | 0 |  |
| Poulos, 2007, Australia | Clinical Information Management System | Observational, Before and After | low | Multi-site study |
| Cho, 2011, South Korea | Consultation Management System | Observational, retrospective Before and After | low |  |
| Gray, 2015, Canada | CPOE | Observational, Retrospective cohort study | low |  |
| Westbrook, 2009, Australia | CPOE | Observational, Two-stage retrospective study | low |  |
| Mekhjian, 2002, USA | CPOE | Observational, retrospective Before and After | low |  |
| Stone, 2009, USA | CPOE | Observational, Retrospective and prospective Before and After | low |  |
| Baumlin, 2010, USA | ED Clinical IS | Observational, Before and After | low | Observation for post data |
| Bushelle, 2017, USA | EHR | Observational | low | Multi-site study |
| Daniel, 2010, USA | EHR | Retrospective observational study | low |  |
| Kennebeck, 2012, USA | EHR | Observational, retrospective Before and After | low |  |
| Pyron, 2019, USA | EHR | Observational, Retrospective Before and After | low | Multi-site study |
| Ward, 2014, USA | EHR | Observational, longitudinal study | low |  |
| Ward, 2014, USA | EHR | Observational, prospective Before and After | low |  |
| Risko, 2014, USA | EHR | Observational, prospective Before and After | low |  |
| Vartak, 2009, USA | EHR, CPOE, Patient Tracking System | Observational, prospective Before and After | low |  |
| Feblowitz, 2017, USA | Electronic Document System | Observational, Retrospective | low |  |
| Furukawa, 2010, USA | EMR | Observational, Longitudinal study | low | Multi-site study |
| Inokuchi, 2015, Japan | EMR | Crossover RCT | high |  |
| Jung, 2020, South Korea | EMR | Observational, retrospective Before and After | low |  |
| Mohan, 2013, Australia | EMR | Observational, retrospective Before and After | low |  |
| Nelson, 2017, Australia | EMR | Ethnographic qualitative design | low |  |
| Tall, 2015, USA | EMR | Observational, retrospective Before and After | low |  |
| Alamo, 2012, Uganda | EMR | Observational, retrospective Before and After | low |  |
| Sicotte, 2016, Canada | EMR | Observational, retrospective Before and After | low |  |
| Crilly, 2015, Australia | Patient Admission Prediction System | Observational, Prospective Before and After | low | Multisite study |
| McLeod, 2010, Canada | Patient flow Dashboard System | Observational, retrospective Before and After | low |  |
| Clark, 2013, Australia | Patient flow Dashboard System | Improvements stated, no figures provided | 0 |  |
| Clark, 2014, Australia | Patient flow Dashboard System | Observational, Retrospective Before and After | low |  |
| Arenson, 1988, USA | Patient scheduling system | Expert opinion | 0 |  |
| Rolls, 2020, Australia | Patient Tracking System | Observational, retrospective Before and After | low |  |
| Mathews, 2014, USA | Patient Tracking System | Observational, Before and After | low |  |
| Borowitz, 1996, USA | Patient Tracking System | Observational, Before and After | low |  |
| Ewing, 2017, USA | Patient Tracking System | Observational | low |  |
| Jensen, 2004, USA | Patient Tracking System | Expert opinion | 0 |  |
| Maloney, 2007, USA | Patient Tracking System | Observational, Before and After | low |  |
| Welch, 2007, USA | Patient Tracking System | Observational, Retrospective analysis | low |  |
| Tran, 2016, Australia | Prescription Management System | Observational, prospective Before and After | low |  |
| Dackiewicz, 2000, USA | Radiology Information System | Observational, Comparative study | low | Compare HIS intervention with traditional intervention |
| Nitrosi, 2007, Italy | Radiology Information System | Observational, retrospective Before and After | low |  |
| Nazar, 2016, England | Referral Management System | Multi-method study | low | Quantitative and qualitative methods |
| Gomes, 2011, Portugal | Surgery Information System | Observational | low |  |
| Li, 2013, Taiwan | Workflow Management System | Observational, retrospective Before and After | low |  |
