# Supplementary material for: Understanding the impacts of health information systems on patient flow management: A systematic review across several decades of research
Source: PLoS One. 2022 Sep 12;17(9):e0274493. doi: 10.1371/journal.pone.0274493 (PMC9467348; doi:10.1371/journal.pone.0274493)
Supplement: Attachment — Submitted filename: Response to Reviewers.docx [file pone.0274493.s007.docx]

Dear Dr Yong-Hong Kuo,

First of all, we would like to express our gratitude to you and the reviewers for having spent your precious reviewing and giving valuable feedback on our systematic review. Your feedback and comments have helped us improve the quality of our manuscript.

In this letter, we provide a detailed description of how we addressed your and the reviewers’ feedback and incorporated them into the revised version of the manuscript.

I. **Editor’s comments**
   1.1 *Please ensure that your manuscript meets PLOS ONE's style requirements, including those for file naming.*

- We set all file names according to Plos One’s guidelines on naming.
- We changed font-size and format of the 1^st^-level heading to 18 Bold, the 2^nd^-level to 16 Bold, and the 3^rd^-level to 14 Bold.
- We changed the titles of included tables and figures to Bold.
- We changed “figure” to “fig” for in-text citation of figures.
- We moved the references to right after the main text.
- We used “et al.” for references with more than 6 authors.
- Based on Plos One’s guideline for references, we added DOIs and PMID to all references where applicable.
- We added a list of supporting documents below the references
  1.2 *When you resubmit, please ensure that you provide the correct grant numbers for the awards you received for your study in the ‘Funding Information’ section.*
- We checked this information with our program coordinator and head of graduate school as well as in my study contract with my university. After all the checks, we can confirm that the awards I received does not have any grant number. Hence, we have not changed this.
  1.3 *Please remove any funding-related text from the manuscript and let us know how you would like to update your Funding Statement*
- We have removed all funding information in the manuscript. The funding statement was kept as follows:

"QDN was supported by a Ph.D. scholarship jointly funded by the Monash University Graduate Research Industry Partnership (GRIP) program and by Eastern Health. The Funders of this work did not have any direct role in the design of the study, its execution, analyses, interpretation of the data, or decision to submit results for publication."

- The above text was entered in Plos One’s submission system.
  1.4 *Please complete your Competing Interests on the online submission form to state any Competing Interests. If you have no competing interests, please state "The authors have declared that no competing interests exist."*
- We have changed the text in the Competing Interests section to the following: "The authors have declared that no competing interests exist."
  1.5 *Upon re-submitting your revised manuscript, please upload your study’s minimal underlying data set as either Supporting Information files or to a stable, public repository and include the relevant URLs, DOIs, or accession numbers within your revised cover letter.*
- We uploaded all of the 6 supporting files as our review’s minimal underlying data set. The URLs and DOIs of these supporting files are included below:
  - S1_File: <https://doi.org/10.26180/20291850.v1>
  - S2_File: <https://doi.org/10.26180/20291862.v1>
  - S3_File: <https://doi.org/10.26180/20291886.v1>
  - S4_File: <https://doi.org/10.26180/20291904.v3>
  - S5_File: <https://doi.org/10.26180/20291895.v2>
  - S6_File: <https://doi.org/10.26180/20291913.v1>
  1.6 *Please review your reference list to ensure that it is complete and correct. If you have cited papers that have been retracted, please include the rationale for doing so in the manuscript text, or remove these references and replace them with relevant current references. Any changes to the reference list should be mentioned in the rebuttal letter that accompanies your revised manuscript. If you need to cite a retracted article, indicate the article’s retracted status in the References list and also include a citation and full reference for the retraction notice.*
- We have checked all of the references in our manuscript and can confirm that there are no retracted publications in the list of references of our review. Some changes in our list of references were made in this revised version as follows:
- We added the DOIs and PMIDs of the included references when available.
- We removed the following references from our list of references:

Vissers JM. Patient flow-based allocation of inpatient resources: a case study. European journal of operational research. 1998 Mar 1;105(2):356-70.

DeLone WH, McLean ER. The DeLone and McLean model of information systems success: a ten-year update. Journal of management information systems. 2003 Apr 1;19(4):9-30.

The reason for these removals is that we decided to remove the text discussing the patient flow measures under Section 6.1 in response to the comment 2.2 and 3.2 related to the repetition in our manuscript. This removed two in-text citations of the above references.

- We added the following reference to our list of references because we found that it was missing in the previous version.

Mathews KS, Corso P, Bacon S, Jenq GY. Using the red/yellow/green discharge tool to improve the timeliness of hospital discharges. The Joint Commission Journal on Quality and Patient Safety. 2014 Jun 1;40(6):243-AP3

II. **Reviewer 1’s comments**
    2.1 *In the "4. Method", the sub-titles seem to have typos. The titles should be "4. X", rather than "1. X".*

- We corrected the sub-sections to 4.1, 4.2, etc. as suggested by the reviewer.
  2.2 *The "6. Discussion" contains much information. Please make it more concise and use some sub-titles or sub-sections to construct this section in a more logical manner.*
- We added the following sub-titles to reflect the content in “6. Discussion” to divide it into logical themes.

6.1 Summary of the key findings

6.2 Strength and limitations

6.3 Implications for patient flow management practice

6.4 Implications for future research

- We removed some of the text deemed redundant or repetitive in this section.
  2.3 *The authors listed some previous HIS papers' practical effects without these HISs' underpinning technologies. These HISs' technical supports should also be considered in this review.*
      *Such that, which company / what database technologies provide this specific HIS service. What does the UI present / How does this HIS work in the investigated papers.*
- We highly appreciate this feedback and believe that this recommendation is very relevant to make our review more interesting and valuable. In response to this, we added a supporting table in S3_File that lists all of the information system interventions of the included studies. We also re-visited all of the included studies in our review and tried to extract the technical aspects of the included information system interventions. Unfortunately, many of the included studies in our review did not contain technical details of the interventions. The few studies that contain some technical descriptions make it infeasible to tabulate the database technologies, UI presentation, HISs’ underpinning technologies or how the HIS work in a meaningful way. We completely agree that this lack of technical descriptions limits our understanding of the impacts of these HIS interventions on patient flow management, and this is a limitation of the included studies. We acknowledged this limitation in Section 6 and recommended future research to overcome this limitation.
  2.4 *Adding more figures and tables will make this review more logical and clearer.*
      *For example, the "5. Results" section presented the reviews with various sub-sections. Each sub-section can have a corresponding table, which has columns like, "paper title", "year", "HIS type", "impact", etc.*
- We added a new table (Table 5) to the Section 5 to provide an overview of our result section.
- We also believe that our Fig.2 is an interesting visualisation of the impact of HIS on different areas of the whole care continuum.
  2.5 *This review paper is a "systematic review across several decades of research". Therefore, it could be better if the systematic logic of this review paper can be highlighted. Why this review is systematic? What sub-systems or specific sub-domains does this review study. What are the changes in HISs during these decades? What are the HISs' differences among different countries?*
- We added a text under Table 4 to describe changes in the application of HIS in patient flow management practice over time and the differences between countries. We believe the addition of this text together with Table 4 will provide the audience with more complete information.
- In regard to the question “Why this review is systematic?”, we believe this question is related to our method of conducting this systematic review. If this is the case, we provided a detailed description of our method in Section 4. In this section, we specified how we searched for publications including a systematic search strategy, how we selected relevant studies, extracted and analysed the information including an analytical framework which was used for our review.
  2.6 *The authors also mentioned the influences of the COVID-19 pandemic. Did COVID-19 affect the selected HIS papers in this review? Are these HISs before COVID-19, or after COVID-19?*
- We reviewed our discussion on this point and can confirm that:
  - The HIS interventions in the included studies were before COVID_19.
  - The reason why we mentioned the pandemic is because it may be an additional complicating factor that may be needed to be considered in future research.
  2.7 *Please present the review through more figures, tables, and various pictures, rather than pure text. The figures can make this review more straightforward, and deliver more information.*
- We believe this comment and comment 2.4 are similar and so we added a new table to Section 5.

III. **Reviewer 2’s comments**
     3.1 *Can the authors please review the PLOS guidelines for manuscript submission and revise the contents of the manuscript accordingly. Particularly in question are the "objective, research question" segments. Additionally, the lack of line numbers makes it difficult to give comments.*

- We have added line numbers to the current version of the manuscript.
- We have re-visited PLOS One’s guidelines for formatting and revised our manuscript accordingly.
- We added introductory text to Section 3 to relate our research questions to the whole review.
  3.2 *There is significant overlap in content included in the results and discussion* *sections. Suggest revision.*
- We found that there were major repetitions in the text in Section 5 and Section 6 of our review. Therefore, we removed these major sections as follows:
  - Section 6.1: We removed the discussion on the type of HIS interventions because the information was described in Section 5.
  - Section 6.1: We removed the discussion on the patient flow measures and the Donabedian model because it repeated Section 5.2

Once again, we would like to thank you and the reviewers for your feedback and comments. We believe that by addressing your concerns and incorporating your feedback, our systematic review has improved considerably. Although we have tried our best to address all of your concerns, there may be places in our manuscript requiring further corrections. If necessary, we would be happy to make further corrections. We look forward to hearing from you soon.

Yours Sincerely

Quy Nguyen

Corresponding author
